# Distinct Subdivisions in the Transition Between Telencephalon and Hypothalamus Produce Otp and Sim1 Cells for the Extended Amygdala in Sauropsids

**DOI:** 10.3389/fnana.2022.883537

**Published:** 2022-05-12

**Authors:** Alek H. Metwalli, Antonio Abellán, Júlia Freixes, Alessandra Pross, Ester Desfilis, Loreta Medina

**Affiliations:** ^1^Department of Experimental Medicine, University of Lleida, Lleida, Spain; ^2^Lleida Biomedical Research Institute’s Dr. Pifarré Foundation (IRBLleida), Lleida, Spain

**Keywords:** extended amygdala, preoptic area, subpreoptic area, paraventricular hypothalamus, glutamatergic neurons, chicken, lizard

## Abstract

Based on the coexpression of the transcription factors Foxg1 and Otp, we recently identified in the mouse a new radial embryonic division named the telencephalon-opto-hypothalamic (TOH) domain that produces the vast majority of glutamatergic neurons found in the medial extended amygdala. To know whether a similar division exists in other amniotes, we carried out double labeling of Foxg1 and Otp in embryonic brain sections of two species of sauropsids, the domestic chicken (*Gallus gallus domesticus*), and the long-tailed lacertid lizard (*Psammodromus algirus*). Since in mice Otp overlaps with the transcription factor Sim1, we also analyzed the coexpression of Foxg1 and Sim1 and compared it to the glutamatergic cell marker VGLUT2. Our results showed that the TOH domain is also present in sauropsids and produces subpopulations of Otp/Foxg1 and Sim1/Foxg1 cells for the medial extended amygdala. In addition, we found Sim1/Foxg1 cells that invade the central extended amygdala, and other Otp and Sim1 cells not coexpressing Foxg1 that invade the extended and the pallial amygdala. These different Otp and Sim1 cell subpopulations, with or without Foxg1, are likely glutamatergic. Our results highlight the complex divisional organization of telencephalon-hypothalamic transition, which contributes to the heterogeneity of amygdalar cells. In addition, our results open new venues to study further the amygdalar cells derived from different divisions around this transition zone and their relationship to other cells derived from the pallium or the subpallium.

## Introduction

The telencephalon is considered the most complex and divergent brain region and includes centers and networks that play key roles in regulating goal-directed behaviors based on contextual information, experience, motivation, and emotion (Striedter, 2005, 2016). Understanding the location of this region within the brain Bauplan, its major divisions and subdivisions, and the location and phenotype of neurons derived from each fundamental morphogenetic unit are critical to disentangling its structural and functional organization. Studying the development of this region in different vertebrates is also essential to better understand its evolution and to distinguish between conserved, divergent, and convergent evolutionary patterns. According to the prosomeric model, the telencephalon is derived from the dorsal most part of the secondary prosencephalon (Puelles and Rubenstein, 2003, 2015). In particular, it is derived from a territory that expresses the transcription factor Foxg1 from very early developmental stages in all vertebrates (Xuan et al., 1995; Toresson et al., 1998; Dou et al., 1999; Wilson and Rubenstein, 2000; Zhao et al., 2009). Inactivation of Foxg1 leads to severe hypoplasia of the telencephalon, which points to a critical role of this transcription factor in telencephalic development (Xuan et al., 1995; Dou et al., 1999; Martynoga et al., 2005; Manuel et al., 2010, 2011). During development, this territory divides into two major compartments: (1) a pallium, which expresses the transcription factors Emx1/2, Pax6, and Tbr1/2 (among others) (Fernandez et al., 1998; Puelles et al., 2000; Bishop et al., 2002; Muzio and Mallamaci, 2003) and produces the glutamatergic neurons of the cerebral cortex and part of the amygdala (Hevner et al., 2001; Gorski et al., 2002); and (2) a subpallium that expresses the transcription factors Gsx1/2, Dlx1/2/5/6, and Nkx2.1 (among others) (Eisenstat et al., 1999; Sussel et al., 1999; Puelles et al., 2000; Yun et al., 2001; Xu et al., 2008; Long et al., 2009; Wang et al., 2011) and produces mostly GABAergic neurons for the ventral telencephalon, including the basal ganglia and the centromedial-extended amygdala (Stühmer et al., 2002; Lindtner et al., 2019) as well as a subpopulation of GABAergic interneurons that migrate tangentially to the pallium (Anderson et al., 2001; Marín and Rubenstein, 2001; Nadarajah and Parnavelas, 2002; Nery et al., 2002). Pallial and subpallial telencephalic divisions, with similar molecular profiles, have been identified in different vertebrates (for example, Fernandez et al., 1998; Puelles et al., 2000; Bachy et al., 2002; Abellán and Medina, 2009; Abellán et al., 2009; Moreno et al., 2009; Mueller and Wullimann, 2009; Osório et al., 2010; González et al., 2014). Until recently, the telencephalon was thought to be bounded ventrally by a hypothalamic division that expresses the transcription factors Otp (Orthopedia) and Sim1/2 (Drosophila single-minded gene homologs), named the supraopto-hypothalamic domain (SPV) or simply the paraventricular domain (Acampora et al., 2000; Puelles and Rubenstein, 2003, 2015), which is highly conserved in vertebrates (Caqueret et al., 2005; Bardet et al., 2008; Moreno et al., 2012; Domínguez et al., 2013, 2015; Herget et al., 2014; Santos-Durán et al., 2016; López et al., 2022). Otp and Sim1/2 are essential for the development of basically all neurons of the paraventricular and supraoptic nuclei, including all neuroendocrine cell types (Michaud et al., 1998; Acampora et al., 2000; Caqueret et al., 2005). However, this view has been recently challenged based on the discovery of an overlapping expression area between Foxg1 and Otp in the transition between the telencephalon and the hypothalamus in zebrafish (Affaticati et al., 2015) and mice (Morales et al., 2021). In mice, this frontier region is a distinct radial histogenetic division that produces cells coexpressing both transcription factors, and this division was called the telencephalon-opto-hypothalamic (TOH) domain because it gives rise to parts of the extended amygdala, eye vesicle, and paraventricular hypothalamus (Morales et al., 2021). The newly-defined TOH was previously included in the dorsal part of the paraventricular hypothalamic region (Puelles et al., 2012; Díaz et al., 2015; Ferran et al., 2015). However, based on its expression of Foxg1 in both progenitor cells and mantle, this Foxg1/Otp coexpressing sector was proposed to rather represent the ventral most division of the telencephalon (Morales et al., 2021). Interestingly, this division produces a large subpopulation of glutamatergic neurons of the medial extended amygdala, previously thought to originate in the hypothalamus (García-Moreno et al., 2010). The TOH domain and the central part of SPV also appear to produce minor subpopulations of cells that invade tangentially the telencephalic subpallium and pallium (Garcia-Calero et al., 2021; Morales et al., 2021, 2022).

As noted above, an overlapping area between the Foxg1 and Otp expression domains was recently found in the developing forebrain of zebrafish (Affaticati et al., 2015), which resembles the mouse TOH domain (Morales et al., 2021). However, the study on zebrafish did not analyze the coexpression in cells of the Foxg1/Otp overlapping area and did not study its relationship to the extended amygdala. Moreover, data on the overlap of Foxg1 and Otp in other vertebrates are missing, making it unclear whether the TOH domain is a conserved, fundamental compartment of the vertebrate forebrain or whether it appeared only during the evolution of particular lineages. The aim of this study was to investigate the existence of a TOH domain, coexpressing Foxg1 and Otp, in the frontier between the telencephalic subpallium and the paraventricular hypothalamus in two sauropsids: the domestic chicken (*Gallus gallus domesticus*) and the long-tailed lacertid lizard (the large Algerian Psammodromus, *Psammodromus algirus*). In addition, we aimed to evaluate if the TOH domain produces Foxg1/Otp coexpressing cells for the extended amygdala in sauropsids. In mice and chickens, the transcription factor Sim1 is expressed in the alar hypothalamus overlapping Otp expression (Michaud et al., 1998; Acampora et al., 2000; Caqueret et al., 2005), and at least in mice, a population of Sim1 cells appears to reach the amygdala (Garcia-Calero et al., 2021). Based on these findings, we also analyzed the expression of Sim1 in combination with Foxg1 in chickens. We compared these results to the expression of VGLUT2, which marks glutamatergic neurons in the pallium and paraventricular hypothalamus and is present in cell subpopulations of the extended amygdala in chickens (Abellán and Medina, 2009; Abellán et al., 2009).

## Materials and Methods

### Tissue Collection and Fixation

In this study, we used chicken and lizard embryos. The chicken embryos (*N* = 37) were obtained from fertilized eggs of the *Gallus gallus domesticus* (Leghorn) and were incubated in a draft-free incubator at the humidity of 50–60% until the desired Hamburger and Hamilton (HH) stages (1992), from HH34-35 (equivalent to 8 incubation days or E8) to HH44 (E18).

The lizard embryos (*N* = 10) were extracted from fertilized eggs of *Psammodromus algirus* (Linnaeus, 1758) (Sauria: Lacertidae), which were incubated at 26–27°C and 70% humidity until the desired stage. In order to obtain them, pregnant females were captured during late spring in the mountains near Madrid (under permission from Dirección General del Medio Ambiente of Madrid, reference numbers: 10/193908.9/11, 10/179954.9/12, and 10/068493.9/16) and in L’Albí (Lleida) (permission from Direcció General de Medi Natural i Biodiversitat of Catalonia, reference number SF/282-284). The lizards were kept in captivity at the Animal Facilities of the University of Lleida until they laid eggs. Following this, females were kept in captivity during a short recovery period and then released at the same point of capture.

All the animals were treated according to the regulations and laws of the European Union (Directive 2010/63/EU) and the Spanish government (Spanish Law 32/2007; Royal Decrees 53/2013 and 118/2021) for the care and handling of animals in research. The experimental protocols used were approved by the Committees of Ethics for Animal Experimentation and Biosecurity of the University of Lleida (reference no. 6127 and CEEA 08-02/19), as well as that of the Catalonian government (reference no. CEA/9960_MR1/P3/1).

After dissection, the brains were fixed at 4°C in 4% paraformaldehyde (PFA, pH 7.4) for 24 h, washed with 0.1 M phosphate-buffered saline (PBS, 0.1 M, pH 7.4), and cryopreserved at −20°C in a hybridization buffer [50% formamide molecular (Thermo Fisher Scientific, Waltham, MA, United States), 1.3% standard saline citrate (pH 5), 5 mM ethylenediaminetetraacetic acid (pH 8) (Sigma-Aldrich, Darmstadt, Germany)], 1 mg/ml of yeast tRNA (Sigma-Aldrich), 0.2% Tween-20, and 100 μg/ml of heparin (Sigma-Aldrich), completed with water (free of RNAase and DNAase; Sigma-Aldrich), until further use.

### *In situ* Hybridization

Previously obtained brains were embedded in a 4% low-melt agarose matrix and sectioned in frontal, sagittal, and horizontal planes using a vibratome (Leica VT 1000S, thickness 80–100 μm). While sectioning, the samples were maintained in the PBS at 4°C. Prehybridization was performed in the hybridization buffer for 2 h at 58°C. After this step, the sections were then hybridized in the hybridization buffer [50% formamide molecular (Thermo Fisher Scientific), 10% dextran sulfate (Sigma-Aldrich), 1 mg/ml of yeast tRNA (Sigma-Aldrich), 0.2% Tween-20, 2% Denhardt solution (Sigma-Aldrich), and a 10% salt solution completed with water free of RNAase and DNAase (Sigma-Aldrich)], containing the selected riboprobe (0.5–1 μg/ml depending on the probe) overnight at 63°C for the chickens and 58°C for the lizards (information on genes and cDNA templates to prepare riboprobes is shown in Table 1). After the hybridization, the sections were washed abundantly first with a 1:1 mix of MABT (1.2% maleic acid, 0.8%; NaOH, 0.84% NaCl, and 0.1% Tween-20) and hybridization buffer at 58°C (2 washes of 20 and 15 min) at room temperature (RT), and then with MABT for 2 h at RT while exchanging the MABT buffer every 15 min. The sections were then blocked in a blocking solution [10% blocking reagent (Roche Diagnostics, Basel, Switzerland) and 10% sheep serum (Sigma-Aldrich) in MABT] for 4 h at RT, and then incubated with an alkaline-phosphatase (AP)-coupled anti-digoxigenin antibody (Roche; diluted 1:3,500) overnight at 4°C, followed by washing with MABT. Signaling was revealed by incubation with nitroblue tetrazolium/5-bromo-4-chloro-3-indolyl phosphate (NBT/CIP, Roche). The sections were then fixed with 4% PFA, and selected sections were further processed for immunohistochemistry.

**TABLE 1:** Genes and cDNAs employed for *in situ* hybridization.

Gene name	GenBank accession no.	Insert size	Obtained from
Chicken Orthopedia Homeobox (cOtp; here referred as Otp)	AY651764.1	851 bp (1–851)	Caqueret et al., 2005
Chicken single-minded homolog 1 (cSim1; here referred as Sim1)	BU292855.1 (ChEST579o9.1 BBSRC ChickEST database)	874 bp (33–907)	BBSRC ChickEST database (Boardman et al., 2002)
Chicken vesicular glutamate transporter 2 (vGlut2)	XM_001234284 (ChEST45417 BBSRC ChickEST database)	619 bp (4,098–3,479)	BBSRC ChickEST database (Boardman et al., 2002)
Lizard Orthopedia Homeobox (Otp)	Newly cloned from tissue of *Psammodromus algirus*, using degenerate primers based on the genome of *Anolis carolinensis* (GenBank Assembly ID GCA_000090745.1). Forward primer: GGSCTSCAGTCYCACCTCTA Reverse primer: GAAGCTCATGGAGACYGTGT The cloned fragment was inserted in: pCRII Vector (Invitrogen/Thermo Fisher Scientific Inc.)	186 bp	Produced at the Molecular Marker Service (SCSIE) of the University of Valencia (Spain)

### Immunohistochemistry

Free-floating sections were processed for immunohistochemistry to detect either Otp or Foxg1 (as described in previous publications of our laboratory, such as Vicario et al., 2017; Desfilis et al., 2018; Morales et al., 2021) (see details on antibodies in Tables 2, 3). Some of the sections were processed for single immunohistochemistry, while other sections were processed for immunohistochemistry after previous hybridization. Briefly, the sections were treated for peroxidase deactivation (incubation in 0.1 M Tris buffer containing 1% H2O2 and 2% methanol) for 30 min under gentle shaking, washed with 0.1 M PBS, permeabilized with PBST (0.1 M PBS, 0.3% Triton X-100) for 20 min, and followed by incubation with a blocking solution containing 10% normal goat serum and 2% bovine serum albumin, for 1 h at RT. Subsequently, the sections were incubated in the primary antibody (rabbit anti-Foxg1 IgG diluted 1:1,000 in the blocking buffer for the chickens and 1:4,000 for the lizards) for 48–72 h under gentle shaking at 4°C. Then, the sections were rinsed with 0.1 M PBS for 10 min at RT, followed by incubation in the secondary antibody (goat anti-rabbit IgG, biotinylated, diluted 1:200 in PBST) for 2 h at RT. The sections were then washed in PBS for 10 min and incubated with the avidin-biotin complex for 1 h at RT under gentle shaking. Finally, the sections were rinsed with 0.1 M Tris buffer and incubated in diaminobenzidine (DAB, SIGMAFAST, 3,3′ diaminobenzidine tablets; Sigma-Aldrich) diluted in a Tris-buffered solution also containing urea and H2O2 until the signal of sufficient quality was achieved. The reaction was stopped with several rinses in a 0.1 M Tris buffer. Then, sections were placed in 4% PFA before being mounted. Afterward, the sections were dehydrated with ethanol (sequential steps in 70, 96, and 100%), cleared in xylol, and finally coverslipped with a permount medium (Thermo Fisher Scientific).

**TABLE 2:** Primary antibodies.

Primary antibodies				
Antibody name	Type	Immunogen	Dilution	Manufacturer and reference
Sheep anti-digoxigenin-AP (alkaline phosphatase conjugate), Fab fragments	Polyclonal	Digoxigenin	1:3500	Roche, 11093274910
Sheep anti- digoxigenin-POD (peroxidase conjugate), Fab fragments	Polyclonal	Digoxigenin	1:200	Roche, 11207733910
Rabbit anti Foxg1, IgG	Polyclonal	Synthetic peptide corresponding to Human FOXG1, aa 400 to the C terminus, conjugated to keyhole limpet hemocyanin	1:1000	Abcam antibodies, ab18259, AB_732415 RRID
Rabbit anti Otp	Polyclonal	Synthetic peptide of 325 aa at the C terminal region of human OTP	1:1000	Antibodies-online, ABIN183823

**TABLE 3:** Secondary antibodies.

Secondary antibodies			
Antibody name	Type	Dilution	Manufacturer and reference
Goat anti-rabbit IgG	Biotinylated	1:200	Vector, Ref BA-1000
Donkey anti-rabbit Alexa Fluor 488	Fluorescent	1:500	Invitrogen, Ref A11034

### Fluorescence *in situ* Hybridization

The tissue was permeabilized with PBST for 30 min, followed by prehybridization for 2 h at 58°C. Sections were hybridized overnight at 63°C in a hybridization buffer containing the riboprobe (0.5–1 μg/ml depending on the probe). Then, the sections were thoroughly washed with the prehybridization buffer for 30 min at 58°C, followed by a wash in a sodium-citrate buffer (SSC, 0.2 M, pH 7.5) for 10 min at RT. Next, sections were treated with the peroxidase deactivation buffer described above, followed by washes in TNT (10% Tris, pH 8, 0.1 M; 0.9% NaCl; 0.05% Tween-20) for 15 min. Subsequently, the samples were treated with the blocking buffer (2% blocking reagent and 20% sheep serum in TNT) for 2 h under gentle shaking. After the blocking, sections were incubated in the primary antibody anti-DIG POD (sheep anti-digoxigenin peroxidase conjugated antibody diluted 1:200 in the blocking buffer; Roche) overnight under gentle shaking at 4°C in the dark. The next day, samples were thoroughly rinsed with a TNT buffer, then treated with a TSA working solution (tyramide conjugated to Cy3, diluted 1:50, and freshly prepared before the reaction; PerkinElmer, Waltham, MA, United States) for 10 min in the dark. The reaction was stopped by a thorough wash with the 0.1 M Tris buffer. Sections were maintained in dark, in the 0.1 M Tris buffer, at 4°C until further use (for immunofluorescence).

### Immunofluorescence

Sections previously processed for indirect FISH were permeabilized with PBST and treated with blocking buffers as described in the section on immunohistochemistry. Then, the sections were incubated with the anti-Foxg1 antibody (diluted 1:1,000 in blocking buffer) for 72 h under gentle shaking at 4°C in the dark. Subsequently, the sections were thoroughly washed as described before and incubated with a secondary fluorescent antibody (donkey anti-rabbit coupled to Alexa 488, 1:500; Invitrogen) for 2 h at RT in the dark. Then, the sections were thoroughly washed with PBS and maintained in the dark before mounting. Finally, they were coverslipped with an antifading mounting medium (VectashieldHardset Antifade mounting medium with DAPI; Vector Laboratories, Ingold Road Burlingame, CA, United States).

### Image Acquisition

Digital microphotographs from conventional *in situ* hybridization and immunohistochemistry were obtained with a Leica microscope (DMR HC; Leica Microsystems GmbH, Germany) equipped with a Zeiss Axiovision Digital Camera (Carl Zeiss, Germany). Serial images from a fluorescent material were taken with a confocal microscope (Olympus FV1000; Olympus Corporation, Japan). Selected digital immunohistochemical images were adjusted for brightness and contrast with Corel PHOTO-PAINT 2012 (Corel Corporation, Canada), while fluorescent images were adjusted and extracted using Olympus FV10-ASW 4.2 Viewer (Olympus Corporation). Finally, figures were mounted using CorelDraw 2012 (Corel Corporation).

## Results

### Otp vs. Fogx1

**FIGURE 1:**
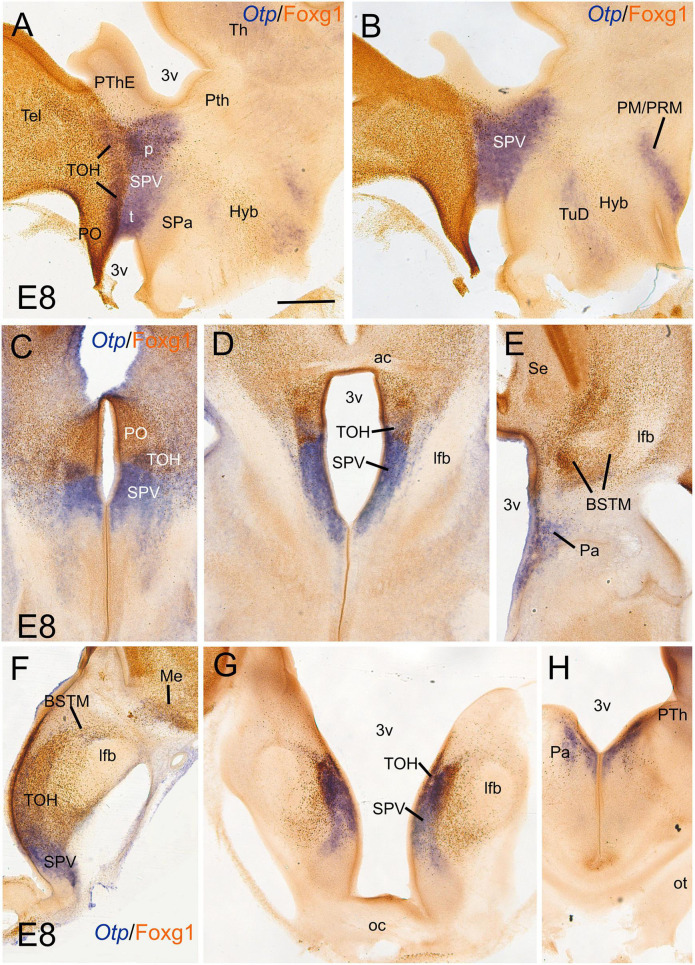
Chromogenic double labeling of Otp and Foxg1 in the chicken forebrain. **(A,B)** Sagittal, **(C–E)** horizontal, and **(F–H)** frontal sections of the chicken embryonic forebrain, at E8, hybridized for Otp (blue color) and immunostained for Foxg1 (brown color). Note the overlapping expression of both transcription factors in the telencephalon-opto-hypothalamic (TOH) domain, just dorsal to the SPV core. See text for more details. For abbreviations, see list. Scale: bar in **(A)** = 200 μm (applies to all).

**FIGURE 2:**
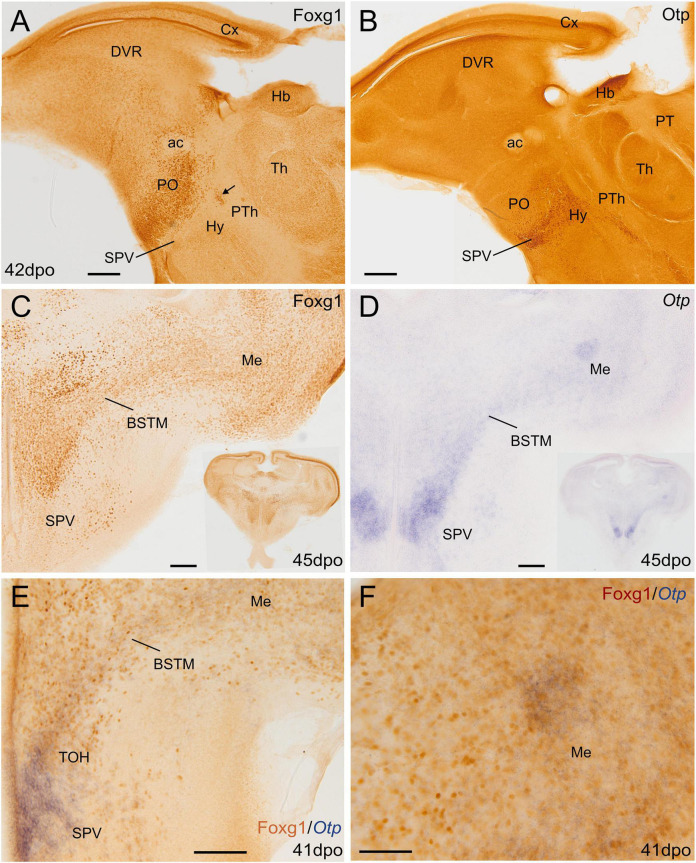
Chromogenic single and double labeling of Otp and Foxg1 in the lizard forebrain. **(A,B)** Sagittal and **(C–F)** oblique horizontal adjacent sections of the lizard embryonic forebrain, 41–45 days post-oviposition, comparing the expressions of Otp and Foxg1. Sections in panels **(A–C)** are immunostained for either Foxg1 or Otp (brown color). The section in panel **(D)** is hybridized for Otp (blue color). The section in panel **(E)** is double labeled for Otp (by *in situ* hybridization, blue) and Foxg1 (by immunohistochemistry, brown). Note the overlapping expression of both transcription factors in the TOH domain, covering a cell corridor that extends from part of BSTM to the medial amygdala (Me). Panel **(F)** shows a detail of a patch of overlapping expression in the Me. The arrow in **(A)** points to a patch of Foxg1 cells in the paraventricular hypothalamic nucleus (in the SPV core). See text for more details. For abbreviations, see list. Scale bars: **(A,B)** = 200 μm; **(C–E)** = 100 μm; **(F)** = 50 μm.

We first compared the expression domains of Otp and Foxg1 in the forebrain of chicken and lizard embryos, in sagittal, horizontal, and frontal sections, by conducting double labeling as follows: for Otp, we carried out chromogenic *in situ* hybridization to detect the mRNA and was followed by immunohistochemistry to detect Foxg1 protein (Figures 1, 2; Otp signal is seen in dark blue, and Foxg1 immunoreactivity is visualized in brown). Similar findings were observed in the chickens (Figure 1) and lizards (Figure 2). In the sagittal and horizontal sections, we observed an Otp signal in the paraventricular hypothalamus (the so-called supraopto-paraventricular hypothalamic domain or SPV), while Foxg1 immunoreactivity was high in the telencephalon (Figures 1A–E, 2B,D). However, we observed an overlapping area between both the expression domains, which we named the TOH (Figures 1A,C,D, 2E; also, compare Figure 2A with Figure 2B and Figure 2C with Figure 2D). The TOH domain extended through peduncular (p) and pre-peduncular or terminal (t) prosomeric subdivisions (Figure 1A). At peduncular level, it included a part of the medial bed nucleus of the stria terminalis (BSTM; Figures 1A,E, 2E) and formed a cell corridor that extended laterally into the medial amygdala (Me) (Figures 2D–F). At terminal level, the TOH included a subpreoptic (SuPO) region located ventral to the subpallial preoptic (PO) area (Figure 1C). The ventricular zone (vz) of the TOH domain was better appreciated in horizontal and frontal sections and showed Foxg1 expressing cells adjacent to Otp expressing periventricular cells (Figures 1D,F,G). The peduncular and pre-peduncular prosomeric divisions of the TOH domain were sequentially observed in frontal sections, from anterior (topologically dorsal) to posterior (topologically ventral) levels (Figures 1F,G): at an anterior section level, only the terminal, subpreoptic subdivision was observed (Figure 1F), while in subsequent posterior sections, the peduncular TOH domain was seen (Figure 1G), followed by the paraventricular hypothalamic domain (in SPV core, Figure 1H). In these sections, terminal and peduncular parts of the TOH domain were ventrally flanked by the Otp-rich/Foxg1-poor SPV core domain (Figures 1F–H). The latter included the primordium of the paraventricular nucleus (Figures 1E,H) and at least part of the supraoptic nucleus. In addition to a major population of Otp cells, the SPV core was observed to contain mostly dispersed Foxg1 immunoreactive cells (Figures 1E,H).

**FIGURE 3:**
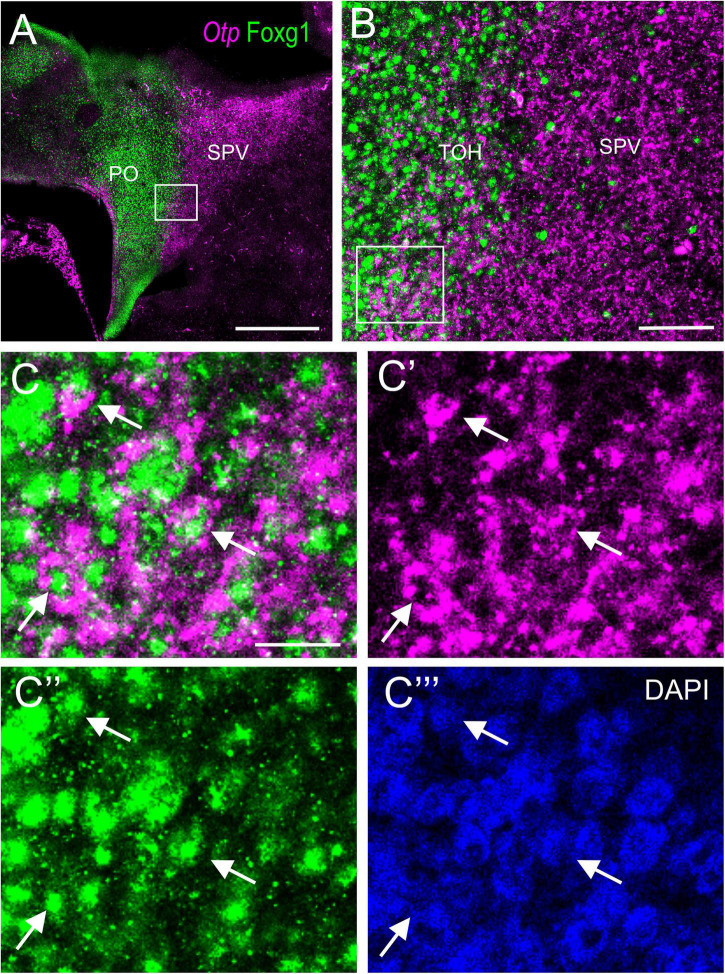
Fluorescent double labeling of Otp and Fogx1 in chicken. **(A)** Sagittal section of the chicken embryonic forebrain, at E8, hybridized for Otp (magenta color) and immunostained for Foxg1 (green color). Note the overlapping expression of both transcription factors in the TOH domain, just dorsal to the SPV core. **(B)** Detail of the area squared in **(A)** showing coexpression of Otp and Foxg1 in many cells of the TOH domain. Panels **(C–C′′′)** show the area squared in B at higher magnification (**C**: merged magenta/green channels; **C′**,**C′′**: separate magenta and green channels; **C′′′**: DAPI staining). Arrows point to a few examples of double-labeled cells. See text for more details. For abbreviations, see list. Scale bars: **(A)** = 320 μm; **(B)** = 80 μm; **(C)** = 20 μm (applies to **C–C′′′**).

**FIGURE 4:**
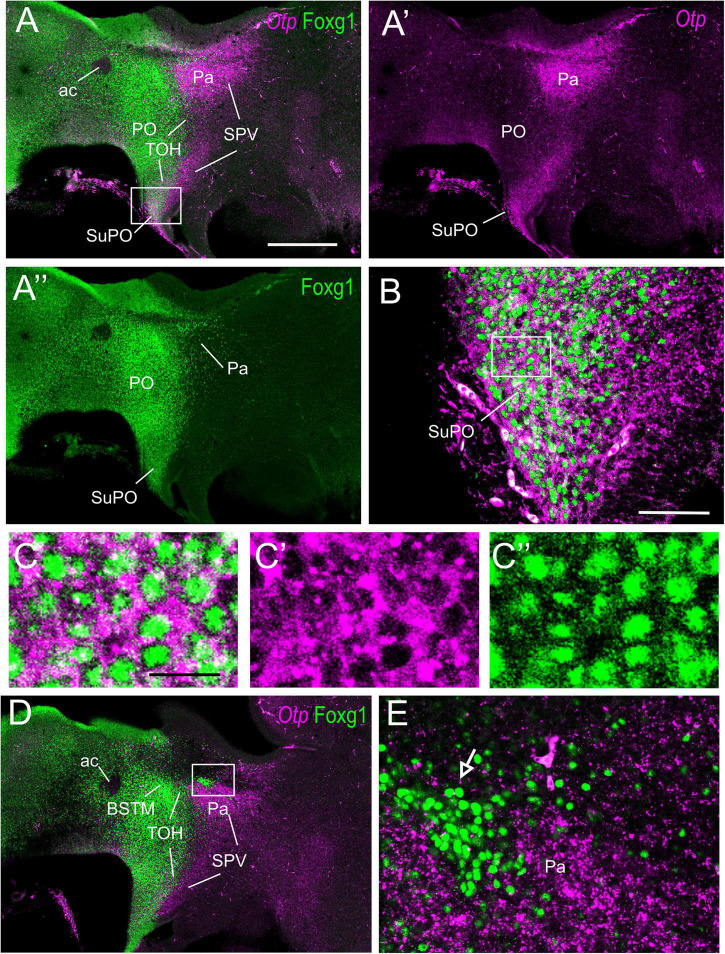
Fluorescent double labeling of Otp and Fogx1 in chicken. **(A–A′′,D)** Sagittal sections of the chicken embryonic forebrain, at E8, hybridized for Otp (magenta color) and immunostained for Foxg1 (green color) (merged and separate channels are shown in **A–A′′**). **(B)** Detail of the area squared in **(A)**, showing coexpression of Otp and Foxg1 in many cells of the subpreoptic (SuPO) area, at the terminal prosomeric part of the TOH domain. Panels **(C–C′′)** show the area squared at B at higher magnification (**C:** merged magenta/green channels; **C′,C′′**: separate magenta and green channels). **(E)** Detail of the area squared in **(D)** showing a patch of Foxg1 cells (pointed with an arrow) inside the paraventricular nucleus (in the peduncular SPV core). See text for more details. For abbreviations, see list. Scale bars: **(A)** = 320 μm (applies to **A–A′′,D**); **(B)** = 80 μm; **(C)** = 20 μm (applies to **C–C′′**).

**FIGURE 5:**
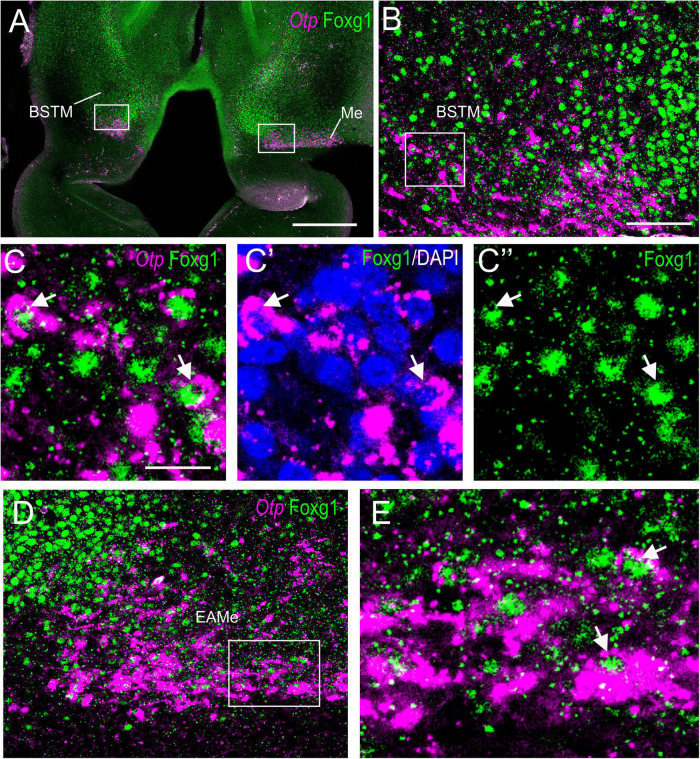
Fluorescent double labeling of Otp and Fogx1 in chicken. **(A)** Horizontal section of the chicken embryonic forebrain, at E8, hybridized for Otp (magenta color) and immunostained for Foxg1 (green color). **(B)** Detail of the area squared in **(A)** (left side) showing coexpression of Otp and Foxg1 in many cells of the ventral part of BSTM (using topological references according to the prosomeric model). Panels **(C–C′′)** show the area squared at B at higher magnification (**C:** merged magenta/green channels; **C′**: magenta channel with DAPI; **C′′**: green channel). Arrows point to examples of double-labeled cells. **(D)** Detail of the area squared in **(A)** (right side) showing coexpression of Otp and Foxg1 in many cells of the medial extended amygdala (a cell corridor that extends from BSTM to the Me). Panel **(E)** shows the area squared at higher magnification (example of a double-labeled cell pointed with an arrow). See text for more details. For abbreviations, see list. Scale bars: **(A)** = 320 μm; **(B)** = 80 μm (applies to **B,D**); **(C)** = 20 μm (applies to **C–C′′,E**).

**FIGURE 6:**
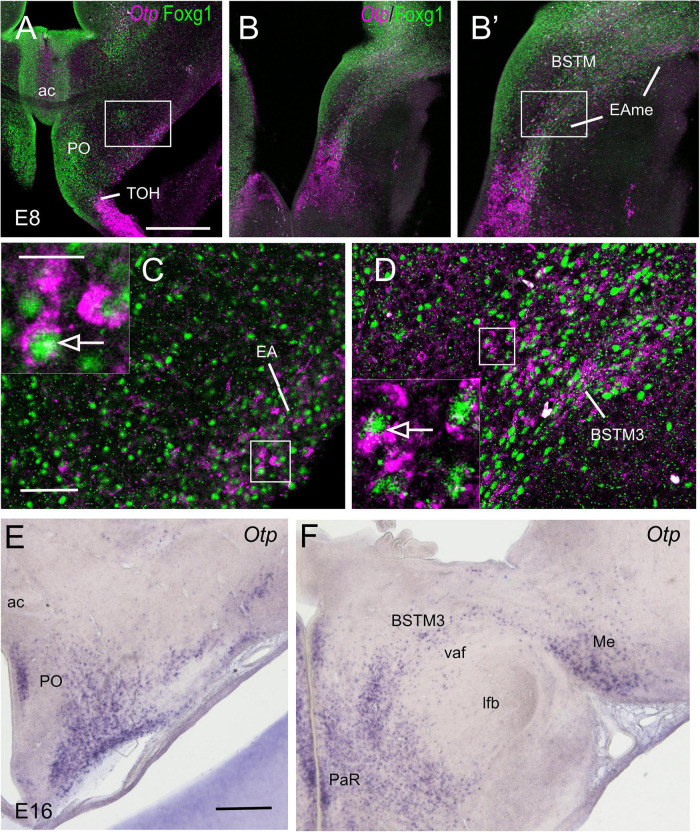
Single and double labeling of Otp and Fogx1 in chickens. **(A,B)** Frontal section of the chicken embryonic forebrain (commissural and post-commissural levels), at E8, hybridized for Otp (magenta color) and immunostained for Foxg1 (green color). Panel **(B′)** is a detail of the cell corridor extending from BSTM to the Me, containing many double-labeled cells. **(C)** Detail of the area squared in **(A)** showing coexpression of Otp and Foxg1 in cells of the preoptic area (PO), including a subpial group that may belong to the extended amygdala (higher magnification detail of double-labeled cells pointed with an arrow in insert). **(D)** Detail of the area squared in **(B)** showing coexpression of Otp and Foxg1 in cells of BSTM (in particular, its BSTM3 subdivision; higher magnification detail of double-labeled cells pointed with an arrow in insert). **(E,F)** Frontal sections of the chicken embryonic forebrain (commissural and post-commissural levels), at E16, hybridized for Otp. Note the corridor of Otp cells extending from BSTM3 to the Me. See text for more details. For abbreviations, see list. Scale bars: **(A)** = 320 μm (applies to **A,B**); **(C)** = 80 μm (applies to **C,D**); **(C)** insert = 20 μm; **(E)** = 400 μm (applies to **E,F**).

**FIGURE 7:**
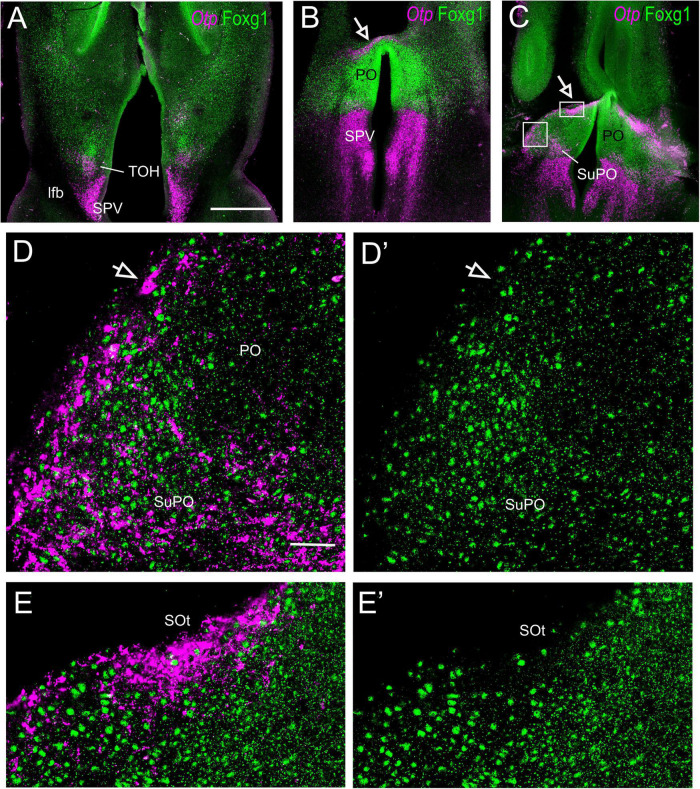
Fluorescent double labeling of Otp and Fogx1 in chickens. **(A–C)** Horizontal section of the chicken embryonic forebrain, at E8, hybridized for Otp (magenta color) and immunostained for Foxg1 (green color). Panel **(A)** is at the peduncular prosomeric level, while **(B,C)** are at terminal levels. Arrows in **(B,C)** point to a stripe of Otp cells in the PO. Panels **(D–E′)** are details of the areas squared in **(C)** (**D,E**: merged channels; **D′,E′**: green channel). Panels **(D,D′)** show double-labeled cells in the SuPO (in the terminal TOH domain), and some Otp single-labeled (arrow) and Otp/Foxg1 double-labeled cells that spread into the PO. Panels **(E,E′)** show a prominent group of Otp single-labeled subpial cells, which seem to correspond to the terminal part of the supraoptic (SOt) nucleus. See text for more details. For abbreviations, see list. Scale bars: **(A)** = 320 μm (applies to **A–C**); **(D)** = 40 μm (applies to **D–E′**).

To know if cells in the TOH domain coexpressed both transcription factors, we carried out doubled fluorescence labeling by way of FISH for Otp combined with immunofluorescence for Foxg1 in chicken forebrain sections (Figures 3–7). In sagittal sections, we observed the Otp/Foxg1 overlapping domain, in the frontier between the subpallium (dorsally) and the SPV core (ventrally) (Figures 3, 4). Observation of the overlapping domain at higher magnification showed a high density of cells apparently coexpressing Foxg1 and Otp (Figure 3B, details of the squared area in Figures 3C–C′′′; Foxg1 transcription factor is seen in green in the cell nucleus, while Otp mRNA is seen in magenta in the cytoplasm). The TOH domain with a high density of Foxg1/Otp cellular coexpression extended from a ventral subdivision of BSTM (at peduncular level; Figure 4D) to the subpreoptic region (at terminal level; Figures 4A,B; a detail of the cells in the subpreoptic region is shown in Figure 4C). These sections also showed ventral dispersion of Foxg1 cells, without coexpression of Otp, into the SPV core (Figures 3B, 4D), and a patch of Foxg1 cells in the paraventricular nucleus (Figure 4D, arrow in Figure 4E). The latter Foxg1 patch or island in the paraventricular nucleus was related to a group of cells that appeared to follow an unlabeled fornix-like fiber tract traveling from dorsal to ventral (Figures 4A,A′′). A similar patch of Foxg1 cells was also seen in the paraventricular nucleus of the lizards (Figure 2A).

Horizontal (Figures 5, 7) and oblique frontal sections (Figure 6) helped to better visualize the contribution of the peduncular TOH domain to the BSTM (Figures 5, 6) and from the terminal TOH domain to the preoptic area (PO) (Figure 7). Regarding the BSTM, our results revealed that this nucleus includes a dorsal part rich in Foxg1 single labeled, and a ventral part rich in Foxg1/Otp coexpressing cells (Figures 5A,B, 6B,B,D′; details of cells in Figures 5C–C′′; inset in Figure 6D). The ventral part of the BSTM rich in double-labeled cells appeared to coincide with a subdivision previously named BSTM3, rich in glutamatergic cells (Abellán and Medina, 2009; see **Figures 14M,N**), but poor in subpallial marker genes such as Nkx2.1, Lhx6, and Lhx7/8 (Abellán and Medina, 2008, 2009). BSTM3 was continuous laterally with a cell stripe of double-labeled cells that reached the Me (Figure 5A-right side, Figures 5D,E, 6B,B′). Thus, the peduncular TOH domain appeared to produce a continuous cell stripe or corridor of double-labeled cells extending from BSTM3 (medially) to a part of the Me (laterally). This corridor continued to be observed in later embryonic stages (Figure 6F). At terminal and peduncular levels, TOH-derived double-labeled cells also appeared to spread tangentially into other parts of the telencephalon, and many were observed near the preoptic subpial surface and more dorsolaterally in a territory previously considered part of the pallial extended amygdala (see Section “Discussion”) (Figures 6A,C,E, 7B,C; details in Figures 7D–E′). Some of the latter cells reached the arcopallium. The TOH-related medial extended amygdala subdivision and other cells near the subpallial subpial surface also contained subpopulations of Foxg1 single- and Otp single-labeled cells. The latter could be better followed in classical horizontal sections (i.e., transversal to the terminal prosomere) (Figure 7C; details in Figures 7D–E′), which showed a particular subpopulation of Otp single-labeled cells that spread dorsally from the terminal part of SPV core, following a subpial path to reach the terminal lamina (arrow in Figures 7B,C). Since these cells only expressed Otp but not Foxg1, they appear to originate in the terminal part of the SPV core. A part of the subpial Otp single labeled cells might correspond to the terminal part of the supraoptic nucleus previously identified in chickens based on its content in vasotocin/mesotocin neurons and named “ventral supraoptic nucleus” (Tennyson et al., 1985; Arnold-Aldea and Sterritt, 1996).

### Sim1 vs. Fogx1

**FIGURE 8:**
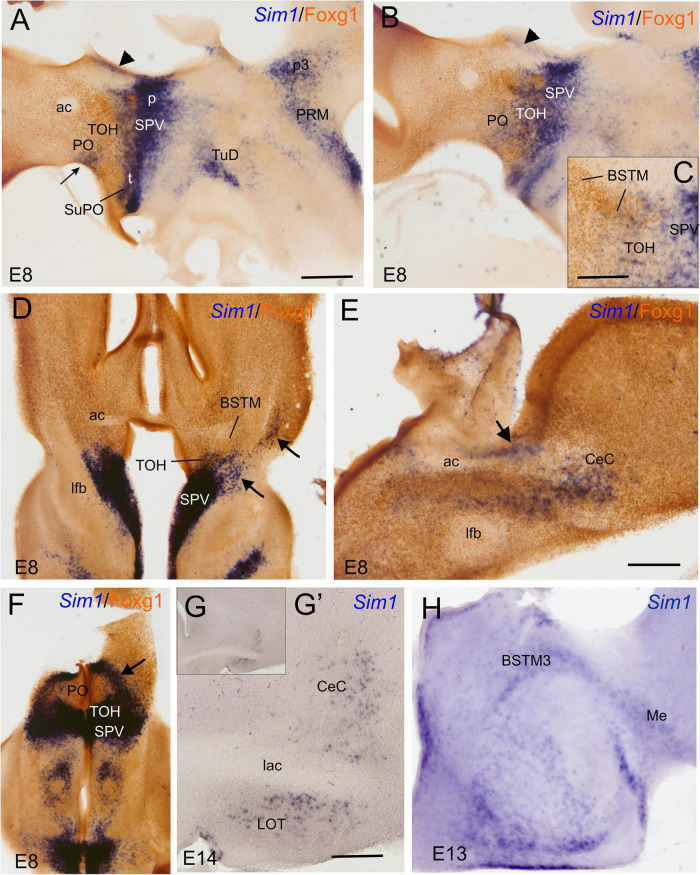
Chromogenic labeling of Sim1 and Foxg1 in the chicken forebrain. **(A–C)** Sagittal, **(D,F)** horizontal, and **(E)** frontal sections of the chicken embryonic forebrain, at E8, hybridized for Sim1 (blue color) and immunostained for Foxg1 (brown color). Note the overlapping expression of both transcription factors in the TOH domain, just dorsal to the SPV core. Panel **(C)** shows a detail of the overlapping area covering part of BSTM (from a section a bit lateral to that seen in **B**). Arrowheads in **(A,B)** show a stream of Sim1 cells spreading dorsally from the SPV core. This stream reaches the BST (pointed with an arrow in **E**). A lateral stream of Sim1 cells (arrow in panel **D**) also reaches the capsular central amygdala and adjacent arcopallium **(E)**. The arrows in **(D,F)** point to streams of Sim1 expressing cells spreading from the TOH and SPV domains to the capsular **(D)** central amygdala or **(F)** the PO. Panels **(G–H)** show details of frontal sections hybridized for Sim1 at the level of the **(G,G′)** central or the **(H)** medial extended amygdala in older embryos (E13 and E14). See text for more details. For abbreviations, see list. Scale bars: **(A)** = 400 μm (applies to **A,B,D,F**); **(C,E)** = 200 μm; **(D)** = 40 μm (applies to **D,E**). **(G′)** = 400 μm (applies to **G′,H**).

We first the analyzed sagittal, horizontal, and frontal sections of a chicken embryonic forebrain doubled labeled for Sim1 (by chromogenic *in situ* hybridization to detect the mRNA) and Foxg1 (by immunohistochemistry to detect the protein) (Figure 8). A strong Sim1 signal was observed in the SPV domain from peduncular to terminal prosomeric levels (Figure 8A), coinciding with the expression of Otp (described in the previous section). Double labeling of Sim1 and Foxg1 showed an overlap of both transcription factors in the TOH domain (Figures 8A,B), similar to that observed with double labeling of Otp and Foxg1. The Sim1/Foxg1 overlapping domain extended from peduncular prosomeric levels (where BSTM3 was found; Figures 8C,D) to terminal prosomeric levels (where the subpreoptic region was located; Figure 8F). At peduncular levels, Sim1 cells were found to spread into the Me (Figure 8H), but also into the central extended amygdala (mostly its lateral part) and the arcopallium (arrow in Figures 8D,G,G′). Some Sim1 cells were also found near the subpial surface of the subpallium, in a territory previously considered part of the pallial extended amygdala (see Section “Discussion”). At terminal levels, Sim1 cells spread in the PO (Figure 8F), following a path similar to that of the Otp cells. In all of these areas, Foxg1 cells were also abundant, but it was unclear whether Sim1 was coexpressed with Foxg1 in some of the cells.

**FIGURE 9:**
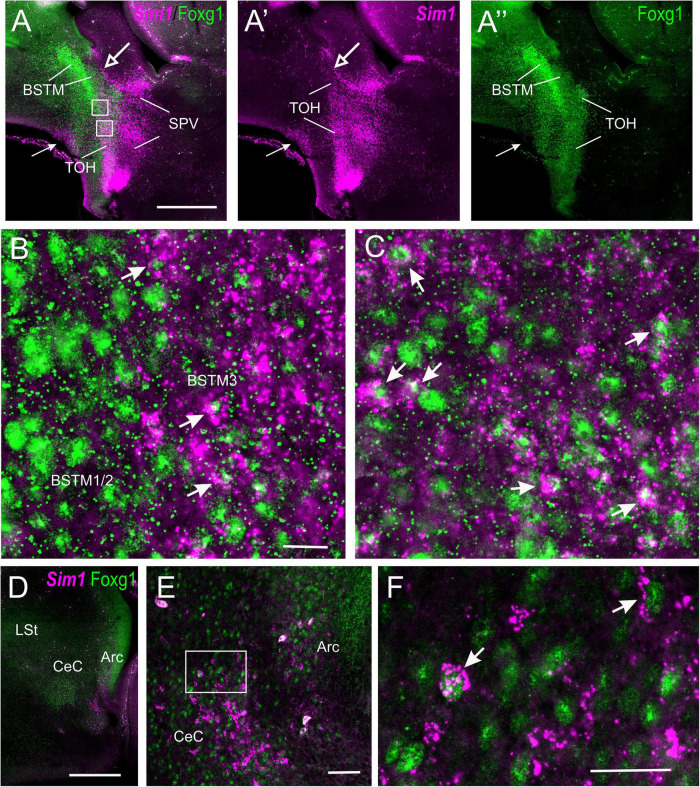
Fluorescent Sim1 and Fogx1 in the chicken forebrain. **(A–A′′,D)** Sagittal sections of the chicken embryonic forebrain, at E8, hybridized for Sim1 (magenta color) and immunostained for Foxg1 (green color) (**A** shows merged channels, and **A′,A′′** show separate channels). Note the overlapping expression of both transcription factors in the TOH domain, just dorsal to the SPV core. Panels **(B,C)** show details of the squared areas in **(A)**, showing coexpression of Sim1 and Foxg1 in many cells of the overlapping area, including BSTM3 (examples of double-labeled cells pointed with an arrow). The large arrow in **(A,A′)** points to a stripe of Sim1 cells, spreading from the SPV core to the vicinity of BSTM. The small arrow in **(A–A′′)** points to Sim1 cells in a subpial position of the PO. Panel **(D)** shows a lateral section, at the level of the capsular central amygdala and adjacent arcopallium. Panel **(E)** is a detail of these areas, and **(F)** shows a higher magnification detail of double-labeled cells in the capsular central amygdala (examples pointed with an arrow). This area also includes some Sim1 single-labeled cells. See text for more details. For abbreviations, see list. Scale bars: **(A)** = 320 μm (applies to **A–A′′**); **(B)** = 40 μm (applies to **B,C**); **(D)** = 320 μm; **(E)** = 40 μm; **(F)** = 20 μm.

**FIGURE 10:**
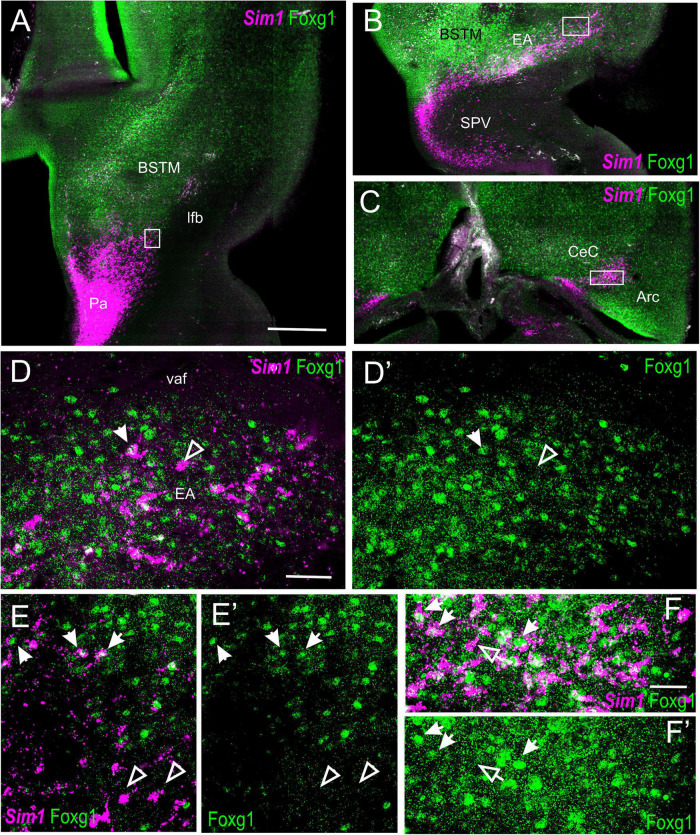
Fluorescent Sim1 and Fogx1 in the chicken forebrain. **(A–C)** Horizontal sections of the chicken embryonic forebrain, at E8, hybridized for Sim1 (magenta color) and immunostained for Foxg1 (green color) (left to right goes from bottom to top). Panels **(D,D′)** are a detail of the area squared in **(B)**, showing Sim1 single-labeled (empty arrow) and Sim1/Foxg1 double-labeled (filled arrow) cells in the medial extended amygdala. Panels **(E,E′)** are a detail of the area squared in **(A)**, showing Sim1 single-labeled (empty arrow) and Sim1/Foxg1 double-labeled (filled arrow) cells in BSTM3 or ventral to it. Panel **(F)** is a detail of the capsular central amygdala from the section shown in **(C)**. Sim1 single-labeled (empty arrow) and Sim1/Foxg1 double-labeled (filled arrows) cells are observed. See text for more details. For abbreviations, see list. Scale bars: **(A)** = 200 μm (applies to **A–C**); **(D)** = 40 μm (applies to **D–E′**); **(F)** = 20 μm (applies to **F,F′**).

To know whether the TOH domain and other parts of the telencephalon contain cells coexpressing Sim1 and Foxg1, we processed sagittal, horizontal, and oblique frontal sections of the chicken embryonic forebrain for double fluorescence labeling by using FISH for Sim1 and immunofluorescence for Foxg1 (Figures 9, 10). An analysis of these sections showed that the TOH domain contains abundant cells apparently coexpressing both transcription factors, resembling the situation of Otp and Foxg1 coexpression (Figures 9A–A′′, details in Figures 9B,C; the Foxg1 transcription factor is seen in green in the cell nucleus, while the Sim1 mRNA is seen in magenta in the cytoplasm). At terminal prosomeric levels, TOH domain-derived double-labeled cells populated the subpreoptic region. In addition, we also observed cells spreading from the TOH domain and SPV core into the PO, where a group of subpial cells, some Sim1/Foxg1 double-labeled, and some Sim1 single-labeled, was found (small arrow in Figure 9A).

**FIGURE 11:**
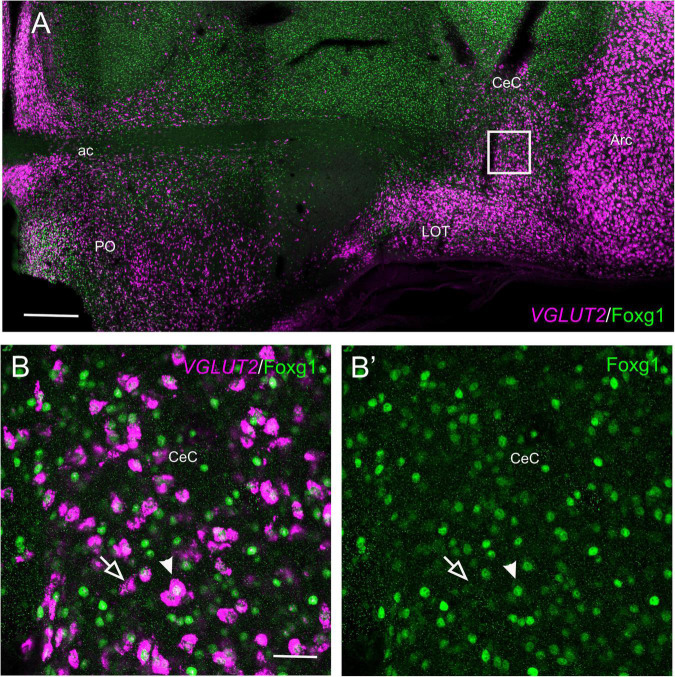
Fluorescent VGLUT2 and Foxg1 in the chicken central extended amygdala. **(A)** Frontal section of the chicken embryonic forebrain (commissural level), at E18, hybridized for VGLUT2 (magenta color) and immunostained for Foxg1 (green color). In addition to those in the pallium, note the presence of a group of VGLUT2 cells in the capsular central amygdala. Panels **(B,B′)** are a detail of this area showing the presence of two types of VGLUT2 cells based on the coexpression of Foxg1 (**B:** merged channels; **B′**: green channel). The filled arrow points to an example of a VGLUT2/Foxg1 double-labeled cell, while the empty arrow points to an example of a VGLUT2 single-labeled cell. See text for more details. For abbreviations, see list. Scale bars: **(A)** = 400 μm; **(B)** = 40 μm (applies to **B,B′**).

In the peduncular prosomere, the Sim1/Foxg1 coexpressing area included BSTM3, which was located just ventral to another part of BSTM (possibly BSTM1/2) that was rich in Foxg1 single labeled cells (Figures 9A,B). However, we also observed another stripe of Sim1 single-labeled cells that extended from the peduncular SPV core to BSTM (large arrow in Figures 9A,A′), thus providing another source of cells for this complex nucleus. Horizontal sections allowed for better visualization of the TOH domain versus SPV core contributions to the extended amygdala (Figure 10). TOH domain-derived double-labeled cells populated ventral parts of the medial extended amygdala, from medial to lateral levels (i.e., from BSTM3 to part of the Me) (Figures 10A,B, details in Figures 10D,D′). SPV core-derived Sim1 single-labeled cells formed a distinct stripe of BSTM (Figure 10A, detail of single-labeled cells pointed with empty arrowheads in Figure 10E), and some were also scattered in other parts of the medial extended amygdala (Figures 10D,D′). Sim1 cells also spread further dorsally reaching the arcopallium. However, in contrast to Otp cells, Sim1 cells reaching the arcopallium seem more abundant, and a subset of Sim1 cells also invades the central extended amygdala, mainly its lateral part (Figures 9D–F, 10C, detailed in Figure 10F). A few cells appear to follow a medial path to reach the BSTL, while the majority of the cells follow a lateral path and penetrate the boundary between the arcopallium and the extended amygdala (Figure 10C), overlapping the previously described intercalated amygdala (Vicario et al., 2014, 2017) from where a few cells spread inside the capsular central amygdala (CeC). Some of the Sim1 cells in the intercalated and capsular central extended amygdala coexpressed Foxg1 (Figures 9E,F,10F), but some Sim1 cells of these areas and many of those in the arcopallium did not (Figure 10F). The distribution of Sim1 cells in the CeC resembled that of glutamatergic cells observed in the same location in a previous publication (Abellán and Medina, 2009; see their **Figure 16F** showing VGLUT2 to be expressed in glutamatergic cells). To better understand the molecular profile of these glutamatergic cells and evaluate their putative origin, we carried out double labeling of VGLUT2 and Foxg1 (both chromogenic and fluorescent) (Figure 11). According to our observations, some VGLUT2 cells of the CeC of the chickens were single-labeled (without Foxg1), although we also observed many VGLUT2/Foxg1 double-labeled cells (Figures 11B,B′). This shows that there are at least two types of glutamatergic cells in the capsular central extended amygdala and suggests that those without Foxg1 must have an extra telencephalic origin, perhaps the same as that of the Sim1 single-labeled cells found in the same location (which appear to derive from the SPV core).

## Discussion

Our results show the existence of a TOH domain, with apparent coexpression of Foxg1 and Otp, in two species of sauropsids that belong to two sister clades of sauropsids: Lepidosauria (including lizards) and Archosauria (including birds). This domain was also previously found in mice (Morales et al., 2021), and it was likely present in the forebrain of stem amniotes. Moreover, an area of Foxg1/Otp overlap resembling the TOH domain has been found in zebrafish (Affaticati et al., 2015), suggesting that it may be a common division in the brain of jawed vertebrates. Like in mice, the TOH division of chickens also expresses Sim1, and our results show an apparent high level of cellular coexpression of Foxg1 and Sim1 in this domain. Given the high abundance of Otp/Foxg1 and Sim1/Foxg1 cells, it is likely that many cells in the TOH domain coexpress Otp, Sim1, and Foxg1. However, considering the observed differences in the presence of Foxg1/Otp versus Foxg1/Sim1 cells in parts of the extended amygdala (as discussed below), it seems that there are at least two different cell populations: (1) cells coexpressing Otp, Sim1, and Foxg1 and (2) cells with Sim1 and Foxg1 but without Otp. The first group seems to be the more abundant (but more studies are needed to quantify) in the TOH domain, including the SuPO and a ventral subdivision of the medial extended amygdala. The second cell group seems to produce a distinct population that tangentially migrates to the central extended amygdala. Groups of Foxg1/Otp and Foxg1/Sim1 cells also spread tangentially into the PO, but it is unclear if they are the same or different cell populations.

As discussed previously (Morales et al., 2021), the TOH domain appears to correspond to the dorsal subdivision of classical SPV (Puelles et al., 2012; Díaz et al., 2015; Ferran et al., 2015). The SPV domain, characterized as a domain of the alar hypothalamus expressing Otp but free of Dlx2/5 and Nkx2.1 and mostly free of Islet1, has been found in different amniotes and anamniotes (Bardet et al., 2008; Morales-Delgado et al., 2011; Moreno et al., 2012; Domínguez et al., 2013, 2015; Herget et al., 2014; Santos-Durán et al., 2016; López et al., 2022). However, based on its expression of the telencephalic Foxg1 transcription factor in the ventricular zone and mantle, we have previously proposed that the TOH domain might be a part of the telencephalon (Morales et al., 2021). This led us to use the term SPV core to refer to the central and ventral parts of classical SPV that are free of Foxg1 in the ventricular zone (Morales et al., 2021). Like in mice, the TOH domain of sauropsids also shows an expression of Foxg1 in the ventricular zone and mantle, while Otp and Sim1 are mostly expressed in postmitotic cells. In contrast, the SPV core ventricular zone is free of Foxg1 expression, and the mantle only contains very few Foxg1 cells. In mice and chickens, the dorsal subdivision of SPV (apparently corresponding to our TOH domain) also differs from the SPV core, because the former does not express Brn2 (Michaud et al., 1998; Caqueret et al., 2005). In amphibians, the ventral part (in topological terms) of SPV expresses the transcription factors Nkx2.2 and Lhx5 (Domínguez et al., 2013). The relationship between these two sectors of amphibian SPV with the TOH domain and SPV core of amniotes requires further investigation, adding Foxg1 as one of the markers. Lhx5 has also been found in the SPV domain of mice and chickens (Abellán et al., 2010), and colocalization of Otp and Lhx5 has been shown in mice (García-Moreno et al., 2010). However, at least in mice and zebra finches, Lhx5 seems to cover a large part (if not all) of SPV and is abundantly found in cells of the TOH-derived part of the medial extended amygdala (Abellán et al., 2010; García-Moreno et al., 2010; Vicario et al., 2017).

In mice, the SPV core gives rise to the main portions of the paraventricular and supraoptic hypothalamic nuclei (Puelles et al., 2012; Morales et al., 2021). Based on previous studies (Arnold-Aldea and Sterritt, 1996; Caqueret et al., 2005; Bardet et al., 2008), combined with our results, this appears to be similar in chickens. In addition, our results on chickens suggest that it also produces minor subpopulations of Otp and Sim1 cells, not coexpressing Foxg1, that migrate tangentially to the PO, extended amygdala, and pallial amygdala/arcopallium. The ventral-to-dorsal dispersion of SPV-domain derived cells (posterior to anterior in topographic terms) was previously suggested in chickens based on an analysis of the distribution of vasotocin/mesotocin immunoreactive cells during development (Arnold-Aldea and Sterritt, 1996). From their site of origin in the anterior (alar) hypothalamus, Arnold-Aldea and Sterritt (1996) described two migratory pathways for these cells: (1) a dorsolateral pathway, which produces the main supraoptic nucleus (named “dorsolateral anterior nucleus, magnocellular component”) by radial migration based on comparison with vimentin, and cells that spread into the BST and the external supraoptic nucleus; (2) a ventrolateral pathway that appears to produce the ventral division of the supraoptic nucleus. Our results partially agree with this suggestion but clarify that the dorsolateral pathway occurs within the peduncular prosomere and that the ventrolateral pathway occurs within the terminal prosomere. In both cases, cells first migrate radially to the subpial surface, and then some appear to take a tangent to continue dorsally (such as those of the external and ventral supraoptic nuclei). Moreover, based on double labeling of Otp/Foxg1 and Sim1/Foxg1, it appears that cells that produce different parts of the supraoptic nucleus do not coexpress Foxg1 and might specifically originate in the peduncular and terminal parts of the SPV core. In addition, the TOH domain gives rise to double-labeled Otp/Foxg1 and Sim1/Foxg1 cells of the extended amygdala and subpreoptic/preoptic areas (some radially, some tangentially, as discussed below). In chickens, TOH and SPV core-derived cells also appear to reach the arcopallium. The presence of these various migration routes from the TOH domain and SPV core toward the PO, extended amygdala, and pallial amygdala agrees with previous descriptions in mice based on Otp/Foxg1 double labeling (Morales et al., 2021, 2022), as well as with descriptions based on Otp and Sim1 single labeling (Garcia-Calero et al., 2021).

### Otp and Sim1 Cells of the Medial Extended Amygdala

As noted above, part of the medial extended amygdala contains numerous cells expressing Otp and Sim1. Otp cells have been previously found in the medial extended amygdala of amniotes and anamniotes (for example, Bardet et al., 2008; Moreno et al., 2010; Abellán et al., 2013; Herget et al., 2014; Biechl et al., 2017; Porter and Mueller, 2020; López et al., 2022), but more data on Sim1 in different species are needed. Our results show that many Otp and Sim1 cells of the medial extended amygdala coexpress Foxg1 and appear to derive from the TOH domain. In the avian BSTM, Otp/Foxg1 and Sim1/Foxg1 cells occupy a ventrolateral division called BSTM3 (included as part of the hypothalamic BSTM in zebra finches; Vicario et al., 2017). In contrast, BSTM1 and 2 are rich in Foxg1 single-labeled cells and seem to correspond to the nuclear subdivisions rich in subpallial cells, such as those expressing Lhx6 (derived from the ventrocaudal pallidal/diagonal domain) and those expressing Shh (derived from the commissural preoptic domain) (Abellán and Medina, 2009; Vicario et al., 2017). While cells of subpallial origin found in BSTM1/2 are GABAergic (Abellán and Medina, 2009), those found in BSTM3 are mostly glutamatergic (Abellán and Medina, 2008, 2009). BSTM also includes subpopulations of cells expressing vasotocin and mesotocin (Aste et al., 1998; Jurkevich et al., 1999; Vicario et al., 2017), and we have previously suggested that these likely originate in SPV (Abellán et al., 2013; Medina et al., 2017) based on the critical role of Otp and Sim1 in the differentiation of these cells (Acampora et al., 2000). However, this needs to be reexamined to discern between contributions of the TOH domain and/or the SPV core, since BSTM does not only include Otp/Foxg1 and Sim1/Foxg1 cells [from the TOH domain, but also Otp and Sim1 single-labeled cells (likely derived from the SPV core)].

In contrast to the clear delimitation of sectors in the avian BSTM and in the mouse Me, the Me of birds is quite small, and cells of different origins are rather intermingled (Abellán and Medina, 2009; Medina et al., 2017; Vicario et al., 2017). At least in the early and middle embryonic stages, Otp/Foxg1 and Sim1/Foxg1 cells of the avian Me form a clear continuum with those of BSTM3, following the ventral amygdalofugal tract. Like that of other amniotes, the avian medial extended amygdala has been involved in social behaviors (Panzica et al., 2001; Xie et al., 2010; Goodson, 2013; Mayer et al., 2017, 2019; Medina et al., 2019). More studies will be required to know more about Otp and Sim1 cells of the medial extended amygdala and their specific role in different aspects of social behavior.

### Sim1 Cells of the Central Extended Amygdala and Adjacent Pallium

We found Sim1 cells in the central extended amygdala and the adjacent pallium of chickens. Regarding the pallium, the finding of Sim1 in the arcopallium and LOT area resembles that of the mouse pallial amygdala, proposed to derive from the SPV core (in particular central SPV; Garcia-Calero et al., 2021). Many Sim1 cells of the chicken pallium did not coexpress Foxg1, which would agree with an extra telencephalic origin in the SPV core. In mice, SPV-derived Sim1 cells appear to populate layer 2 of the nucleus of the lateral olfactory tract after putative long migration through the posterior pole of the pallium to end in the subpallium (Garcia-Calero et al., 2021). In chickens, Sim1 cells may also contribute to populating the LOT nucleus, suggested to be located near the surface, under the end of the lateral branch of the anterior commissure (Puelles et al., 2018, see **Figure 14** in the 2nd edition of the Atlas of the Chicken Brain). In agreement with this proposal, this area receives an olfactory input (Reiner and Karten, 1985) and contains Sim1 cells (present results; Figures 8G,G′). The LOT appears to be part of a larger subpial area rich in glutamatergic cells that is continuous laterally with those in the arcopallium and other parts of the avian pallial amygdala (present results, Figure 11A; Abellán et al., 2009). This area also extends a bit rostral to commissural levels, always close to the pial surface, and has been identified in our previous publications as part of the pallial extended amygdala, a glutamatergic cell corridor extending from the SPV to the pallial amygdala (Abellán et al., 2009, 2010). A comparison of Sim1 with Foxg1 shows that this area contains Sim1/Foxg1 double-labeled and Sim1 single-labeled cells (present results), indicating an external origin of at least some of the cells of this corridor. Moreover, this ample area also contains Otp cells (part of them coexpressing Foxg1, as noted in the results) and other cell types with different molecular profiles and apparent origins, such as those with Lhx5 and Tbr1 derived from the prethalamic eminence (PThE) and those with Lhx9 of pallial origin (as discussed previously by Abellán et al., 2009; see also Abellán and Medina, 2009; Abellán et al., 2010; Vicario et al., 2017; Alonso et al., 2020, 2021). The LOT of mice also appears to include a variety of cell subtypes with different molecular profiles and embryonic origins (discussed by García-López et al., 2008, Garcia-Calero et al., 2021). Sim1 cells are located in its layer 2 (Garcia-Calero et al., 2021), and our previous data have suggested that the mouse LOT also contains and/or is surrounded by scattered Otp cells, which avoid layer 2 (Morales et al., 2021). Thus, Otp and Sim1 cells seem to occupy different layers in this pallial nucleus in mice.

One striking finding of our study is the observation of a group of Sim1 cells in the chicken central extended amygdala. This subpallial complex comprises BSTL and additional subdivisions interposed between BSTL and arcopallium, which include the CeC (Vicario et al., 2014, 2015, 2017). A few Sim1 cells were found in BSTL, but a large subpopulation was found in the CeC. Our results show that some of the Sim1 cells of this capsular area coexpress Foxg1 and some do not. It is unclear whether similar cell populations are present in other vertebrates. In the early embryonic stages, most Sim1cells are found in the intercalated cell area, interposed between the CeC and the arcopallium. The majority of Sim1 cells found in the intercalated area in early stages may be in transit to neighboring areas, including the capsular central extended amygdala since the amount of capsular Sim1 cells seems to increase during subsequent stages and the cells resemble a subset of glutamatergic cells observed in this area (Abellán and Medina, 2009; present results). Based on the presence of a subset of Lhx9 cells in this same location, we have previously proposed that they likely originate in the Lhx9-rich amygdalar pallium (Abellán et al., 2009). However, our results on Sim1 cells suggest that at least some glutamatergic cells of the central extended amygdala originate in the TOH domain and the SPV core and migrate tangentially to the subpallium during development. Further investigation will be required to know the exact origin of the different glutamatergic cells found in the central extended amygdala and their functional relationship to the predominant GABAergic cells found in this same territory. The central extended amygdala of mammals plays a key role in triggering and regulating fear and anxiety responses (Phelps and LeDoux, 2005; Davis et al., 2010). In birds, the BSTL also plays a role in fear and anxiety (Nagarajan et al., 2014; reviewed by Smulders, 2021), but the implication of other parts of the central extended amygdala is unknown. The finding of subsets of glutamatergic cells in the BSTL and the capsular part of the central extended amygdala raises questions on the possible interactions of these glutamatergic cells with the typical GABAergic cells found in this nuclear complex.

### Glutamatergic vs. GABAergic Cells of the Telencephalon

Telencephalic function in the regulation of goal-directed behaviors by contextual information, motivation, and emotions depends on exquisite modulation of glutamatergic and GABAergic networks. Unbalance in these excitatory/inhibitory networks is behind many mental and neurodevelopmental disorders (Gao and Penzes, 2015; Sohal and Rubenstein, 2019). Most telencephalic nuclei and areas contain a mixture of both cell subtypes in different proportions. In the cerebral cortex, basolateral amygdala, and cortical amygdala, the predominant cells are glutamatergic, while in the septum, basal ganglia, and centromedial extended amygdala, the predominant cells are GABAergic (reviewed by Medina and Abellán, 2009, 2012; Moreno et al., 2009). Glutamatergic and GABAergic neurons of the telencephalon are considered to be primarily derived from one of the two major embryonic divisions of the telencephalon, the pallium and the subpallium, respectively (Hevner et al., 2001; Stühmer et al., 2002; Osório et al., 2010). Until now, only minor cell subpopulations were thought to originate outside, in the PThE and alar hypothalamus, and migrate tangentially to the telencephalon during development. Our study challenges this view with the identification of a new embryonic domain, the TOH domain, which expresses Otp, Sim1, and Foxg1, and is located in the ventral most part of the telencephalon. This domain, observed in sauropsids (present results) and mice (Morales et al., 2021), produces a large population of glutamatergic cells for the medial extended amygdala, and subsets of glutamatergic cells for the subpallium and pallium. This new division contributes to the heterogeneity of cells found in the amygdala and other telencephalic areas and opens new venues to further study the relationship of TOH domain-derived cells to other cells derived from the pallium or the subpallium.

## Data Availability Statement

The raw data supporting the conclusions of this article will be made available by the authors, without undue reservation.

## Ethics Statement

The animal study was reviewed and approved by Committees of Ethics for Animal Experimentation and Biosecurity of the University of Lleida (reference no. 6127 and CEEA 08-02/19), as well as that of the Catalonian Government (reference no. CEA/9960_MR1/P3/1).

## Author Contributions

AM processed most of the chicken embryonic brain material (including all early stages of embryonic brains) as part of his Ph.D. research project. AA and AP contributed to the processing. AA produced the additional material from older chicken embryos. AM photographed and prepared many of the study figures and analyzed the material with the help of AA and LM. JF and ED processed the lizard embryonic brains, analyzed the material, and obtained the digital photographs. AM, LM, JF, and ED prepared the figures for the article. AM and LM produced the first draft of the manuscript. All authors revised and approved it.

## Conflict of Interest

The authors declare that the research was conducted in the absence of any commercial or financial relationships that could be construed as a potential conflict of interest.

## Publisher’s Note

All claims expressed in this article are solely those of the authors and do not necessarily represent those of their affiliated organizations, or those of the publisher, the editors and the reviewers. Any product that may be evaluated in this article, or claim that may be made by its manufacturer, is not guaranteed or endorsed by the publisher.

## Abbreviations

3v: third ventricle
ac: anterior commissure
Arc: arcopallium
BST: bed nucleus of the stria terminalis
BSTL: lateral BST
BSTM: medial BST
BSTM1-3: subdivisions 1–3 of BSTM
CeC: capsular central amygdala
DVR: dorsal ventricular ridge
EA: extended amygdala
EAme: medial EA
Hb: habenula
Hy: hypothalamus
Hyb: basal Hy
lac: lateral branch of ac
lfb: lateral forebrain bundle
LOT: nucleus of the lateral olfactory tract
LSt: lateral striatum
Me: medial amygdala
oc: optic chiasm
ot: optic tract
p: peduncular prosomere
p3: prosomere 3
Pa: paraventricular hypothalamic nucleus
PaR: rostral (terminal prosomeric) part of Pa
PM: peri-mammillary area
PO: preoptic area
PRM: peri-retromammillary area
PT: pretectum
PTh: prethalamus
PThE: prethalamic eminence
Se: septum
SOt: terminal prosomeric division of the supraoptic nucleus
SPa: subparaventricular nucleus
SPV: supraopto-paraventricular hypothalamic domain
SuPO: subpreoptic area
t: terminal prosomere
Tel: telencephalon
Th: thalamus
TOH: telencephalon-opto-hypothalamic domain
TuD: dorsal tuberal region
vaf: ventral amygdalofugal tract.
